# Matrine Activates PTEN to Induce Growth Inhibition and Apoptosis in ^V600E^BRAF Harboring Melanoma Cells

**DOI:** 10.3390/ijms140816040

**Published:** 2013-07-31

**Authors:** Hui Jin, Yu Sun, Shuiying Wang, Xiaodong Cheng

**Affiliations:** 1 School of Life Sciences and Technology, Tongji University, Shanghai 200092, China; E-Mail: joycejinhui@tongji.edu.cn; 2 Yue-yang Hospital, Shanghai University of Traditional Chinese Medicine, Shanghai 200437, China; E-Mails: sy060323@hotmail.com (Y.S.); ashikaru@163.com (S.W.); 3 East Hospital, Tongji University, Shanghai 200120, China

**Keywords:** Matrine, cell cycle arrest, apoptosis, ^V600E^BRAF melanoma, PTEN

## Abstract

Here, we report a natural chemical Matrine, which exhibits anti-melanoma potential with its PTEN activation mechanism. Matrine effectively inhibited proliferation of several carcinoma cell lines, including melanoma ^V600E^BRAF harboring M21 cells. Flow cytometry analysis showed Matrine induced G_0_/G_1_ cell cycle arrest in M21 cells dose-dependently. Apoptosis in M21 cells induced by Matrine was identified by Terminal deoxynucleotidyl transferase dUTP nick end labeling (TUNEL) analysis and Annexin-V/FITC staining. Molecular mechanistic study suggested that Matrine upregulated both mRNA level and protein expression level of phosphatase and tensin homolog deleted on chromosome ten (PTEN), leading to inhibition of the PI3K/Akt pathway. Downregulation of phosphor-Akt^ser473^ by Matrine activated p21 and Bax, which contributed to G_0_/G_1_ cell cycle and apoptosis. Besides, Matrine enhanced the PI3K/Akt inhibition effects to inhibit the cell proliferation with PI3K inhibitor, LY2940002. In summary, our findings suggest Matrine is a promising antitumor drug candidate with its possible PTEN activation mechanisms for treating cancer diseases, such as melanomas.

## 1. Introduction

The tumor suppressor gene phosphatase and tensin homolog deleted on chromosome ten (PTEN) is a 47 kDa protein, which was first identified as a candidate tumor suppressor gene in 1997 [1,2]. The PTEN protein has both protein phosphatase and lipid phosphatase activity [3,4]. As a phosphatase, PTEN acts to remove the phosphate in PtdIns(3,4,5)P3 to generate PtdIns(4,5)P2 by counteracting with the phosphoinositide 30 kinase (PI3K), resulting in inhibition of the PI3K/Akt pathway [5]. The PI3K/Akt pathway is a very important cell signal pathway, which is overactive in many cancers, reducing apoptosis and allowing proliferation [6]. Many reports showed activating PTEN is able to downregulate the PI3K/Akt pathway, which led to antitumor potential in cancer treatment [7–9]. Molecular mechanistic studies of PTEN showed activating PTEN induced cell cycle arrest, apoptosis and regulated cell adhesion, migration and differentiation. Besides, PTEN co-acts with several important tumor-relating factors, including p53, Egr-1, NF-κB [10–12], *etc.*, which facilitates the tumor suppressor function of PTEN. These interactions make PTEN exerting unique biochemical functions and playing an important role in tumor suppression.

PTEN is often downregulated in many poorly differentiated cancers, including breast cancer, colon cancer and melanomas [13–15]. Melanoma is estimated to be the fifth most common cancer in males and the sixth most common in females in the United States in 2009 [16], and there were 76,250 estimated new cancer cases reported in the United States in 2012 [17]. Malignant melanoma (MM) is one of the most aggressive malignant tumors, and the incidence of melanoma is increasing sustainedly and rapidly worldwide. Molecular biological studies about melanoma show BRAF mutation is one of the most frequent findings in human melanomas. These mutations cluster in the glycine-rich loop and activation segments of the kinase domain, with a single missense substitution (V600E), identified in over 90% of cases [18]. Intensive analysis suggests loss of PTEN is commonly associated with BRAF mutations in multiple carcinoma diseases [19]. Previous studies showed that loss of PTEN function through mutation or deletion has been observed in up to 70% of melanoma cell lines, and epigenetic silencing of PTEN has been observed in 30%–40% of malignant melanomas [20]. Functional studies reported that in PTEN-deficient melanoma cells, expression of PTEN was able to reduce melanoma tumorigenicity and metastasis [21], implicating PTEN acting as a critical tumor suppressor in melanoma tumorigenesis. However, the molecular mechanisms involved in regulating PTEN functions and details of interactions between PTEN and the PI3K/Akt pathway in melanoma are poorly understood. The potential to regulate PTEN function as novel strategies for melanoma treatment remains largely unknown.

**Figure 1:**
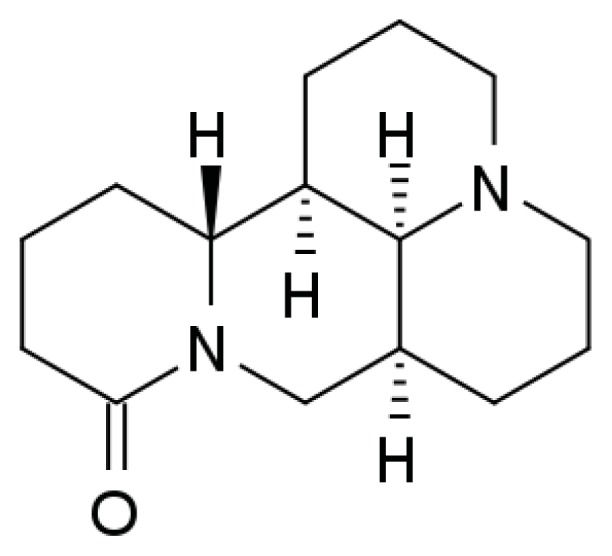
The structure of Matrine.

Matrine (C_15_H_24_N_2_O, Figure 1) is an alkaloid, which is the major active component in the traditional Chinese medicine, *Sophora flavescens* [22]. Matrine has been used widely in China for the treatment of viral [23], hepatitis [24], hepatic fibrosis [25], arrhythmia [26] and skin diseases [27]. In recent years, increasing studies showed Matrine also exhibits antitumor effects by inhibiting proliferation and inducing cell cycle arrest and apoptosis in different cancer cells, including leukemia, gastric cancer, hepatocellular carcinoma, breast cancer and lung cancer. Molecular mechanistic research showed that Matrine regulated tumor regulators, including NF-κB, XIAP, Bcl-2 and Bax, *etc.*, as well as interacted with cell signal pathways, including extracellular signal-regulated kinases (ERK) and Fas/FasL, *etc.* [22,28–34]. However, the anti-tumor potential and underlying mechanism of Matrine still remain largely unknown.

In this study, we evaluated the antitumor potential of Matrine in a ^V600E^BRAF harboring melanoma M21 cells. We found Matrine inhibited the cell proliferation in M21 cells, but did not affect the normal human retinal pigment epithelium cells. Matrine induced cell cycle arrest at the G_0_/G_1_ phase and apoptosis in M21 cells dose-dependently. Matrine activated PTEN to inhibit the PI3K/Akt pathway and, finally, led to p21 and Bax upregulations in M21 cells. These findings suggest that activating PTEN holds promise as practicable strategies for melanoma treatment, and Matrine is a potent candidate for melanoma treatment.

## 2. Results and Discussion

### 2.1. Results

#### 2.1.1. Matrine Exhibited Effective Proliferation Inhibition in M21 Melanoma Cells, but Did Not Affect the Normal Cells

**Figure 2:**
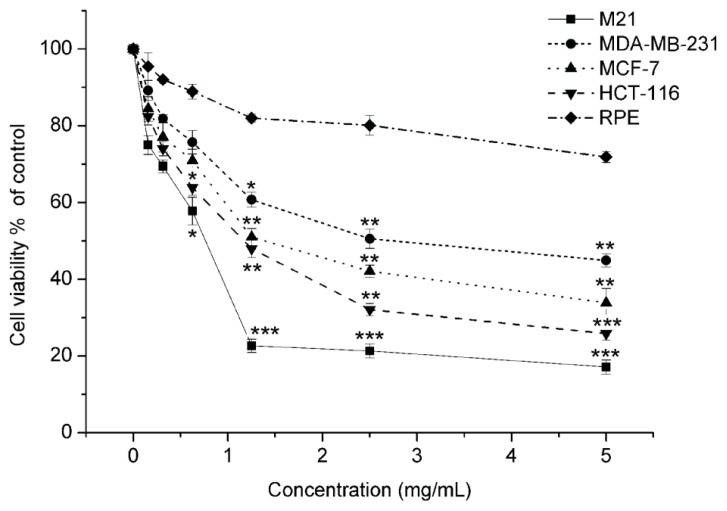
The anti-proliferative activity of Matrine in four carcinoma cell lines and one normal human cell line. Cells were incubated with Matrine as concentrations indicated for 48 h before 3-(4,5-Dimethyl-thiazol-2-yl)-2,5-Diphenyltetrazolium Bromide (MTT) was performed. All experiments were performed at least thrice and independently. Significant differences from untreated control were indicated as *p < 0.05; **p < 0.01; ***p < 0.001.

As shown in Figure 2, Matrine exhibited a dose-dependent cell proliferation inhibition against multiple human cancer cell lines, including tumors from different tissues origins. The calculated IC_50_s were listed in Table 1. The lowest IC_50_ of Matrine was against M21 cells, which suggested its potent anti-proliferation effects in melanoma cells. The IC_50_ against human retinal pigment epithelium (RPE) cells was far beyond the effectual dose in carcinoma cell lines (Figure 2). Since RPE cells were normal cells and from the same lineage as melanoma, the data indicated that Matrine did not affect the proliferation of normal cells. These findings suggested that Matrine effectively inhibited the proliferation of M21 cells without significant cytotoxicity on normal cells.

#### 2.1.2. Matrine Induced G_0_/G_1_ Phase Cell Cycle Arrest Dose-Dependently

**Figure 3:**
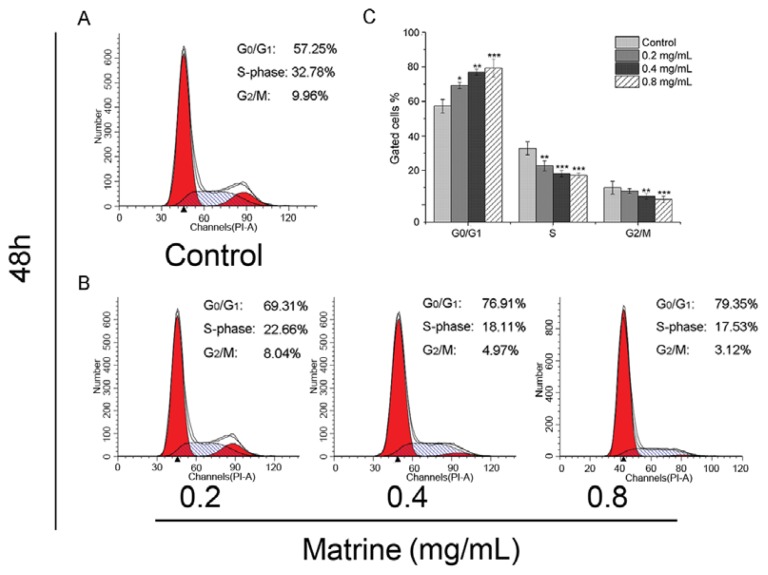
(**A**) Cell cycle distributions in M21 cells as control; (**B**) Cell cycle distributions in M21 cells with Matrine in different concentrations as indicated. M21 cells were treated with Matrine for 48 h before PI staining; (**C**) The analysis of cell cycle distributions in M21 cells with Matrine. All data were expressed as means ± SD of three separate experiments. Significant differences from untreated control were indicated as *p < 0.05; **p < 0.01; ***p < 0.001.

As flow cytometry results showed, Matrine induced significant G_0_/G_1_ accumulation with S and G_2_/M depletion in M21 cells. The proportion at the G_0_/G_1_ phase was 57.25% in the control, whereas the proportion was 69.31% in the cells after treatment with Matrine at the concentration of 0.2 mg/mL (Figure 3B). Seventy-six-point-nine-one percent of 76.91 cells were blocked in the G_0_/G_1_ phase after treatment with Matrine at the concentration of 0.4 mg/mL, which made a significant difference compared to the control (*p* < 0.01) (Figure 3B). At the concentration of 0.8 mg/mL, the proportion of gated cells in the G_0_/G_1_ phase rose to 79.35% consistently. Both the proportions of S and G_2_/M decreased as the concentration increased. The cells with Matrine exposure gated in the S-phase was 17.53% at the concentration of 0.8 mg/mL, which made a significant difference compared to the control (*p* < 0.001). At the concentration of 0.8 mg/mL, the proportion of gated cells in the G_2_/M phase declined to 3.12% (Figure 3B). These findings suggested that Matrine blocked the cell cycle at the G_0_/G_1_ phase in M21 cells dose-dependently (Figure 3C).

#### 2.1.3. Matrine Induced Apoptosis in M21 Cells Dose-Dependently

**Figure 4:**
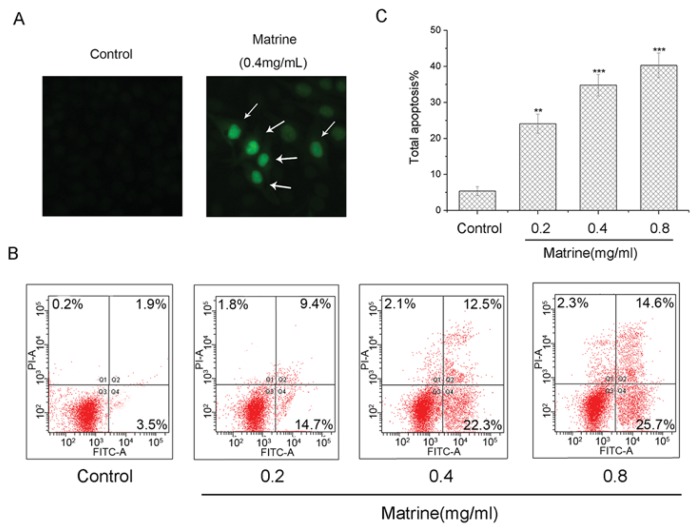
(**A**) Morphologically apoptotic changes in M21 cells with Matrine treatment at concentrations as indicated. M21 cells were treated with Matrine for 48 h before TUNEL staining and photographed; (**B**) Induction of apoptosis in M21 cells with Matrine treatment at concentrations as indicated. M21 cells were treated with Matrine for 48 h before being stained with Annexin V-FITC/PI and flow cytometric analysis; and (**C**) The total apoptosis in M21 cells with Matrine treatment at concentrations as indicated. All data were expressed as means ± SD of three separate experiments. Significant differences from untreated control were indicated as *p < 0.05; **p < 0.01; ***p < 0.001.

To investigate whether Matrine induced apoptosis in M21 cells, cells were treated with Matrine in indicated concentrations before Terminal deoxynucleotidyl transferase dUTP nick end labeling (TUNEL) assay performing. By exposure with Matrine at a concentration of 0.4 mg/mL, we found a significant percentage of M21 cells stained positive after Matrine exposure (Figure 4A). These observations suggested that Matrine induced morphologically apoptotic changes in M21 cells.

Then we performed flow cytometry assay to identify the apoptosis. As shown in Figure 4B, the apoptosis proportion in M21 cells ascended as the concentration increased dose-dependently. The total percentage of apoptotic cells was 24.1%, with Matrine at the concentration of 0.2 mg/mL. As the concentration increased to 0.4 mg/mL, the proportion of total apoptosis was 34.8%. With Matrine treatment at the concentration of 0.8 mg/mL, the total percentage of apoptotic cells was 40.3%, which was significantly different (*p* < 0.001) to the control (Figure 4C). These data suggested that Matrine induced apoptosis in M21 cells dose-dependently.

#### 2.1.4. Matrine Upregulated p21 and Downregulated Cyclin D in M21 Cells

**Figure 5:**
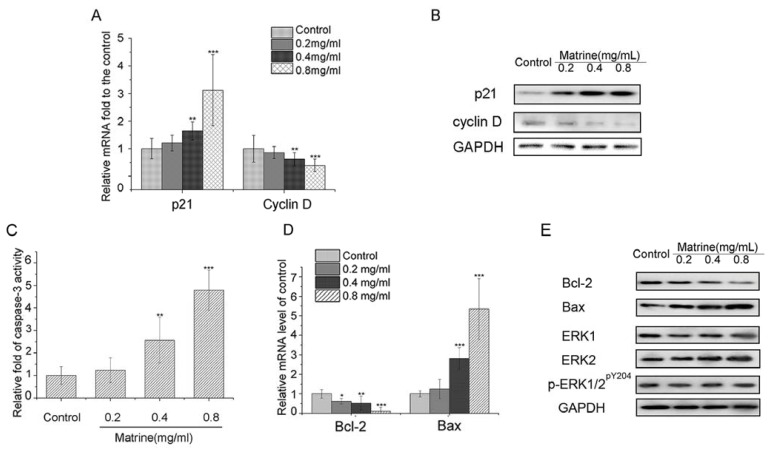
(**A**) Relative mRNA level of p21 and Cyclin D in M21cells with Matrine treatment at concentration as indicated; (**B**) Protein expression of p21 and Cyclin D in M21cells with Matrine treatment at concentration as indicated; (**C**) Relative activity of caspases activity. Cells were incubated with Matrine at concentration, as indicated for 48 h before the activity was tested; (**D**) Relative mRNA level of Bcl-2 and Bax in M21cells with Matrine treatment at concentration as indicated; (**E**) Protein expression of Bcl-2, Bax, ERK1, ERK2 and p-ERK1/2^pY204^ in M21cells with Matrine treatment at concentration as indicated. Cells were incubated with Matrine at concentration as indicated for 48 h before performing Western blotting and real-time PCR. All data were expressed as means ± SD of three separate experiments. Significantly differences from control were indicated as *p < 0.05; **p < 0.01; ***p < 0.001.

To explore the molecular mechanisms, we examined mRNA level and protein expression of G_0_/G_1_ cell cycle regulators, p21, Cyclin D. Real-time PCR data analysis showed that the mRNA level of p21 was upregulated dose-dependently with Matrine. In contrast, the mRNA level of the positive regulator, Cyclin D, were downregulated with Matrine (Figure 5A). Then, we investigated the protein expression of p21 and Cyclin D. As shown in Figure 5B, the protein expression of p21 was promoted, while the expression of Cyclin D was suppressed dose-dependently with Matrine. These results suggested that p21 activation and Cyclin D repression were involved in the molecular mechanisms of cell cycle arrest induced by Matrine.

#### 2.1.5. Both Caspase-3 Activation and Bcl-2/Bax Interference Involved in the Apoptosis Induced by Matrine

To explore whether caspases activation was involved in the induced apoptosis, we examined the activity of caspase-3 in Matrine-treated M21 cells. We found that Matrine significantly activated the activity of caspase-3 (Figure 5C). Compared to the control, the activity of caspase-3 in M21 cells with Matrine at the concentration of 0.4 mg/mL was elevated to 2.57-fold (*p* < 0.01). With Matrine treatment at the concentration of 0.8 mg/mL, the activity of caspase-3 was 4.98-fold of the control (*p* < 0.001) (Figure 5C). These data suggested that Matrine activated the activity of caspase-3 in M21 cells dose-dependently.

Besides, we examined mRNA level of Bcl-2 and Bax in Matrine-treated M21 cells. Interestingly, we found that the mRNA level of Bcl-2 was downregulated, while that of Bax was upregulated by Matrine (Figure 5D). The relative mRNA level of Bcl-2 shrank to 0.09-fold of the control with Matrine at the concentration of 0.8 mg/mL (*p* < 0.001). Nevertheless, the relative mRNA level of Bax increased to 5.48-fold of the control with Matrine at the concentration of 0.8 mg/mL (*p* < 0.001) (Figure 5D). Moreover, Matrine downregulated expression of Bcl-2, while upregulating expression of Bax consistently (Figure 5E). Considering the mitochondrial apoptosis mediated by Bcl-2 and Bax, it is reasonable to believe that Matrine regulated both the caspase and mitochondrion pathway to trigger apoptosis in M21 cells.

#### 2.1.6. Matrine Did Not Affect the Protein Expression of ERK1/2 or Phosphorylation of ERK1/2 in M21 Cells

As inhibition of the MAPK pathway can cause growth arrest and apoptosis in melanoma cell lines, we assessed the effects of Matrine on the expression of key components (ERK1/2) and their activation forms (phosphorylation of ERK1/2) in M21 cells. As shown in Figure 5E, neither the expression of ERK 1 nor ERK 2 was affected by Matrine. Besides, Matrine did not affect the expression of the phosphorylation of ERK1/2 (p-ERK1/2^pY204^). These results suggested that key components of the MAPK pathway were not involved in the anti-tumor effects of Matrine in M21 cells.

#### 2.1.7. Matrine Activated PTEN to the Inhibit PI3K/Akt Pathway in M21 Cells, but Not in A375 Cells

**Figure 6:**
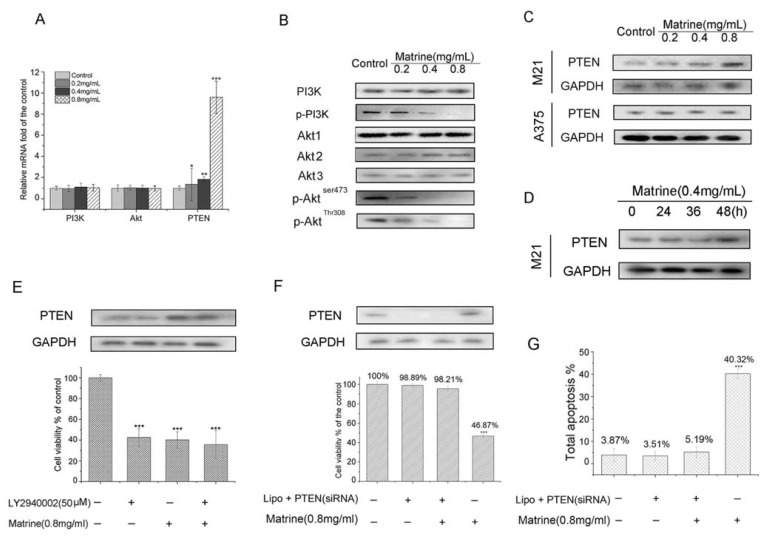
(**A**) Relative mRNA level of PI3K, Akt and PTEN in Matrine treated M21cells. Cells were incubated with Matrine at concentration as indicated for 48 h before performing real-time PCR; (**B**) Protein expression of PI3K, the phosphorylation form of PI3K (p-PI3K), Akt 1, Akt 2 and the phosphorylation form of Akt (p-Akt^ser473^) in M21 cells with Matrine treatment; (**C**) Protein expression of PTEN in M21 cells and A375 cells with Matrine treatment. Cells were incubated with Matrine at concentration as indicated for 48 h before performing Western blotting; (**D**) Protein expression of PTEN in M21 cells with Matrine treatment at the time point as indicated; (**E**) The anti-proliferative activity of Matrine and LY2940002 or combination in M21cells; (**F**) Growth inhibition blocks in PTEN silencing or not M21 cells with Matrine; (**G**) Total apoptosis percentage induction inhibition blocks in M21 cells. All data were expressed as means ± SD of three separate experiments. Significant differences from untreated control were indicated as *p < 0.05; **p < 0.01; ***p < 0.001.

To further explore the molecular mechanisms of Matrine, we assessed the effects on PTEN and the PI3K/Akt pathway of Matrine. Real-time PCR results suggested that Matrine did not affect the mRNA level of PI3K nor Akt, but significantly upregulated the mRNA level of PTEN dose-dependently (Figure 6A). At the concentration of 0.4 mg/mL, Matrine elevated the mRNA level of PTEN to 1.89-fold. When the concentration rose to 0.8 mg/mL, the level was promoted to 9.17-fold compared to the untreated (*p* < 0.001).

Then, we investigated the protein expression of PI3K, the phosphorylation form of PI3K (p-PI3K), Akt1, Akt2 and Akt3 and the phosphorylation form of Akt and PTEN. As shown in Figure 6B,C, Matrine did not affect the expression of PI3K nor that of Akt1, Akt2 and Akt3. Remarkably, Matrine augmented protein expression of PTEN and inhibited the expression of p-PI3K, p-Akt^ser473^ and p-Akt^Thr308^ dose-dependently. Interestingly, Matrine did not affect PTEN expression in A375 cells (Figure 6C). Besides, Matrine upregulated protein expression of PTEN time-dependently (Figure 6D) in M21 cells. Considering the negative control of PTEN in the PI3K/Akt pathway, these results suggested that Matrine upregulated the mRNA level and protein expression of PTEN, which led to the inhibition on phosphorylation of PI3K and Akt.

#### 2.1.8. Matrine Enhanced the Inhibition of PI3K/Akt Pathway to Inhibit Cell Proliferation in M21 Cells

To confirm the effects on the PI3K/Akt pathway with Matrine, a PI3K inhibitor LY2940002 was used to examine the cell viability plus Matrine. As the results show (Figure 6E), the cell viability was 42.57% with LY2940002 solely and that was 40.21% with Matrine solely. The cell viability was 35.69% with Matrine plus LY2940002, which made statistical difference to the control (*p* < 0.001). The data suggested Matrine enhanced the inhibition of the PI3K/Akt pathway to inhibit the cell proliferation in M21 cells.

#### 2.1.9. PTEN Silencing Blocked the Cell Growth Inhibition and Apoptosis Induced by Matrine in M21 Cells

We used a specific PTEN silencing siRNA assay to assess the role of PTEN playing in Matrine-induced cell growth inhibition and apoptosis. We found that the cell growth inhibition and apoptosis were sufficiently blocked in M21 cells with Matrine after PTEN silencing.

As shown in Figure 6F, the cell viability in PTEN silenced M21 cells was 98.21% with Matrine treatment, which made no statistical difference to the untreated ones (*p* > 0.05). Besides, the total apoptosis was 5.19% with Matrine plus PTEN siRNA, whereas the value was 40.32% (*p* < 0.001) without siRNA (Figure 6G). These findings suggested that knockdown of PTEN sufficiently blocked the cell growth inhibition and apoptosis induced by Matrine treatment. These results confirmed that PTEN was a target gene of Matrine and responsible for the cell anti-tumor effects of Matrine in M21 cells.

### 2.2. Discussion

Heterogeneity relating to cancer treatment has been observed in multiple malignant cancers, including melanoma. Both classical cytotoxics and newer targeted approaches that inhibit the pathways dysregulated by alterations in the oncogenes or tumor suppressors are identified as effective therapeutics for cancer treatment. In this study, we reported that a natural product, Matrine, effectively inhibited the proliferation in multiple carcinoma cells, including M21 melanoma cells. Matrine induced G_0_/G_1_ phase cell cycle arrest and apoptosis dose-dependently in M21 cells. That activating PTEN, which led to the PI3K/Akt pathway inhibition by Matrine, suggested that Matrine is a potent candidate for melanoma treatment.

Melanoma is one of the most common skin cancer and increasing sustainedly and rapidly worldwide. Recent progress in the analysis of genetic alterations in melanoma has identified recurrent mutations that result in the activation of critical signaling pathways promoting growth and survival of tumors cells. Alteration in the RAS-RAF-MAP (Mitogen-activated protein kinases) kinase pathway is one of the most commonly observed in melanoma. Most melanoma cell lines with BRAF mutations are dependent upon RAF-MAP signaling and are extremely sensitive to inhibition of the pathway with selective inhibitors [35,36]. These inhibitors induced the proliferation, cell cycle arrest and apoptosis in cell lines and inhibited the tumor cell growth xenografts. Several of them have entered clinical trials for melanoma treatment and exhibited potential in application [37–39]. Besides, the development of immunotherapy with interleukin-2 (IL-2) or interferon (IFN) [40,41] in melanoma treatment seems sometimes to produce durable remission in a small subset of patients. However, for most patients, these treatments are not effective enough. The overall success in melanoma is quite limited.

Matrine is the major active component in the traditional Chinese medicine, *Sophora flavescens*, with a long history. Different studies suggested Matrine inhibited proliferation and induced cell cycle arrest and apoptosis in different cancer cells, as stated in the Instructions section. In this study, we found that Matrine effectively inhibited the proliferation in several carcinoma cell lines, including melanoma M21 cells (Figure 2). The IC_50_ of Matrine against M21 cells was 0.769 mg/mL, which was the lowest among the tested carcinoma cells. Importantly, Matrine did not affect the proliferation of human retinal pigment epithelium (RPE) cells, as the response concentration was far beyond the effectual dose in M21 cells (Figure 2). Meanwhile, Matrine arrested the cell cycle at the G_0_/G_1_ phase and induced apoptosis in M21 cells dose-dependently (Figure 3A–C and Figure 4A–C). Molecular mechanisms showed Matrine upregulated the mRNA level and protein expression of p21 while downregulated those of Cyclin D in M21 cells, which contributed to the G_0_/G_1_ cell cycle by Matrine (Figure 5A,B). Besides, both caspase-3 activation and mitochondrial Bcl-2 and Bax regulation involved in the apoptosis were induced by Matrine (Figure 5C–E). These findings were consistent with other reports about Matrine in other carcinoma cell lines [32,42]. Previously, a study showed Matrine inhibited invasiveness and metastasis of another human malignant melanoma cell line, A375 [43]. Our studies further supported that Matrine inhibited the proliferation in human malignant melanoma cell line M21 without cytotoxicity effects in normal pigment epithelium cells, which indicated the therapeutic potential of Matrine in melanoma treatment.

Recent progress in the analysis of genetic alterations in melanoma has identified recurrent mutations regulating growth and survival of tumors cells, which shed a light on melanoma treatment. Besides alterations in the RAS-RAF-MAP kinase signaling pathway, the PI3/Akt kinase signaling pathway is another commonly altered in melanoma [15,44]. PTEN is a negative regulator of the PI3/Akt pathway. Activation of PTEN leads to inhibition of the PI3K/Akt pathway, which facilitates the tumor cell growth inhibition, cell cycle arrest and apoptosis. However, loss of PTEN function is the most commonly known genetic alteration in the PI3-kinase cascade and is commonly associated with BRAF mutations [45–47]. Besides, increasing reports showed that loss of the PTEN was one important reason to attenuate the anti-tumor effects of RAF inhibitor in melanomas harboring mutant-BRAF [18,48,49]. Nevertheless, the understanding of the potential of PTEN in melanoma still remains largely unknown.

As shown in Figure 5E, Matrine did not affect the protein expression of ERK1/2 or phosphorylation of ERK1/2 in M21 cells. We then investigated another important pathway—the PI3/Akt pathway. We found that Matrine did not affect the mRNA level of PI3K and Akt nor their protein expression (Figure 6A,B). In contrast, Matrine upregulated the mRNA level and protein expression of PTEN in M21 cells dose-dependently and time-dependently (Figure 6A,C,D). Consistently, activation of PTEN induced by Matrine significantly downregulated the phosphorylation level of PI3K and Akt in M21 cells dose-dependently (Figure 6B). These data suggested that Matrine activated PTEN to inhibit the PI3K/Akt pathway dose-dependently. Interestingly, Matrine did not affect PTEN expression in A375 cells (Figure 6C). The reasons for different molecular regulations in PTEN between A375 and M21 with Matrine might be due to different key targets with inherent diversity between cell lines.

To determine the block in the PI3K/Akt pathway with Matrine, we tested the cell viability with a PI3K inhibitor, LY2940002. Expectedly, Matrine enhanced the inhibition of the PI3K/Akt pathway to inhibit cell proliferation in M21 cells, as exhibited in Figure 6E. Moreover, as shown in Figure 6F,G, silencing PTEN sufficiently blocked the cell growth inhibition and apoptosis induced by Matrine in M21 cells. These findings suggested that PTEN was required for the antitumor efficacy of Matrine. A previous paper [50] showed that PTEN expression was observed in some BRAF-mutant human melanoma cell lines following treatment with an MEK inhibitor. In our study, we found that Matrine did not affect the key components of the MAPK pathway (ERK 1, ERK2 and p-ERK1/2) in M21 cells. These results are consistent with reports in another paper [51], which showed that Matrine induced MAPK pathway-independent apoptosis in leukemia U937 cells. Our findings suggested that the PI3K/Akt pathway might play much more important roles than the MAPK pathway in melanoma cells, such as M21. Besides, PTEN-activated PI3K/Akt pathway inhibitors, such as Matrine, might be potent candidates or ideal combinatorial approaches for melanomas, which were not sensitive to MEK inhibitors. These issues need more exploration, and the intensive investigations are on our agenda.

As M21 is a ^V600E^BRAF harboring cell line [52], we reported that Matrine activated PTEN in ^V600E^BRAF melanoma cells, which resulted in cell proliferation inhibition, cell cycle arrest and apoptosis. Besides, these results suggested that activating PTEN is potent and practicable to treat melanoma. In other words, PTEN is a potential therapeutic target in melanoma treatment.

## 3. Experimental Section

### 3.1. Chemicals

Matrine and LY2940002 were purchased from Sigma (M5319, L9908, St. Louis, MO, USA) and diluted in culture media for all experiments *in vitro*. The controls were treated with the same amount of vehicle and the same condition, according to the samples.

### 3.2. Cell Lines and Cell Culture

Cell lines were purchased from Cell Bank of Type Culture Collection of the Chinese Academy of Sciences (Shanghai, China), including breast carcinoma cells (MCF-7 and MDB-MA-231) and human colon carcinoma cells (HCT-116). The human melanoma cell lines (M21) and A375 were purchased from John Wayne Cancer Institute (Santa Monica, CA, USA). The human retinal pigment epithelium (RPE) cells were purchased from ATCC, which were used as normal control. MCF-7, MDB-MA-231, M21 and A375 cells were cultured in Dulbecco’s Modified Eagle Medium (DMEM) medium supplemented with 10% fetal calf serum. HCT-116 were cultured in RPMI1640 medium supplemented with 10% bovine serum. RPE cells were grown in DMEM:F12 (1:1) with 10% fetal calf serum. Penicillin (100 U/mL) and streptomycin (100 U/mL) were added to all kinds of medium. Cells were incubated at 37 °C in a humidified atmosphere of 95% air and 5% CO_2_.

### 3.3. Cell Viability Assay

3-(4,5-Dimethyl-thiazol-2-yl)-2,5-Diphenyltetrazolium Bromide (MTT, Sigma, St. Louis, MO, USA) assay was performed to assess cell proliferation after drug exposure, as described previously [53]. Briefly, cells in 96-well plates were incubated with Matrine at 0, 0.156, 0.3125, 0.625, 1.25, 2.5 and 5 mg/mL for 48 h at 37 °C. After exposure, MTT was added, and absorbance values were collected in an Automated Microplate Reader (Molecular Device, Sunnyvale, CA, USA) at 595 nm. The values were converted to cell numbers according to a standard growth curve of the relevant cell line. The concentration, which reduced cell numbers to 50% relative to vehicle-treated, cells was calculated as IC_50_ by using a Bliss assay.

### 3.4. Flow Cytometric Analysis of Cellular DNA Content

Cells were incubated with Matrine at the concentration of 0.2, 0.4 and 0.8 mg/mL for 48 h. Then harvested, treated and untreated cells were fixed with 70% ice-cold ethanol and were kept at −20 °C overnight. Fixed cells were centrifuged, washed and resuspended in PBS contained in concentration of 50 μg/mL of propidium iodide (PI, P4170, Sigma) and 100 μg/mL of DNase-free RNase at 37 °C for 30 min, as previously reported. Cell cycle was analyzed by using a cell cytometer (Becton Dickinson, Franklin Lakes, NJ, USA) and Cell Quest Pro software (Becton Dickinson, Franklin Lakes, NJ, USA).

### 3.5. Flow Cytometric Analysis of Apoptosis and Necrosis

Apoptosis assessment was performed by using an Annexin-V/FITC apoptosis detection kit (Invitrogen, Carlsbad, CA, USA), as described by the manufacturer’s instructions. Briefly, cells were incubated with Matrine at the concentration of 0.2, 0.4 and 0.8 mg/mL for 48 h. Harvested cells were stained with Annexin-V/FITC and PI in binding buffer for 15 min at room temperature in the dark, as previously reported. The samples were analyzed by FACS can flow cytometry (Becton Dickinson, Franklin Lakes, NJ, USA) to determine the percentage of cells displaying Annexin-V and PI staining.

### 3.6. TUNEL Apoptosis Detection Assay

The TdT-UTP nick end-labeling (TUNEL) method was performed to label the 3′-end of fragmented DNA of the apoptotic cells with a TUNEL Apoptosis Detection Kit, according to the manufacturer’s instructions. Briefly, cells were incubated with Matrine at a concentration of 0.4 mg/mL for 48 h. After treatment, cells were fixed with 4% paraformaldehyde phosphate buffer saline, rinsed with PBS, then permeabilized by 0.1% Triton X-100 for 2 min on ice, followed by TUNEL mixture for 1 h at 37 °C. The labeled TUNEL-positive cells were imaged under a fluorescence microscopy (Nikon, Tokyo, Japan) at 488 nm excitation and 530 nm emission. The cells with green fluorescence were defined as apoptotic cells.

### 3.7. Determination of Caspase Activity

Cells were treated with Matrine at concentration of 0.2, 0.4 and 0.8 mg/mL for 48 h. Caspase-3 activity was determined using a Caspase-3 activity kit (Beyotime), according to the manufacturer’s instructions. Briefly, treated cells were lysed with lysis buffer for 15 min on ice following washing with cold PBS. After incubating, the cell lysates were incubated with 100 μM of enzyme-specific calorigenic substrates at 37 °C for 1 h. Caspase-3 activity was quantified in the samples with a Microplate Reader (Molecular Device, Sunnyvale, CA, USA) at an absorbance of 405 nm. Caspase-3 activity was expressed as the fold of enzyme activity compared to that of synchronized cells.

### 3.8. Real-Time Quantitative Reverse Transcription-PCR Analysis

The cDNA temples were prepared from cells with Matrine by using a FastLane Cell cDNA Kit (Qiagen, Frankfurt, Germany), according to the manufacturer’s recommendations. Real-time PCR was performed by using a QuantiTect SYBR Green PCR Kit (Qiagen, Frankfurt, Germany) with a 7500 Fast Real-Time PCR System (Applied Biosystems, Foster City, CA, USA). Primers were obtained from Shanghai Generay Biotec Co., Ltd (China). The sequences of used primers were as follows: for PTEN, ATT CCC AGT CAG AGG CGC TAT (forward) and TGT GCT GCC CTT CTG TTC AAG (reverse); for PI3K, AGG TTC ATG TGC TGG ATA CT (forward) and TGG GCT CCT TTA CTA ATC TC (reverse); for Akt, ACG ATG AAT GAG GTG TCT GT (forward) and TCT GCT ACG GTG AAG TTG TT (reverse); for p21, TCC CAA ACG CAA AGA CTG (forward) and AGG GTT TGC GTT TCT GAG (reverse); for Cyclin D, AAC TCC CTG CGA AAC ACA C (forward) and TTG AGG GAC GCT TTG TCT G (reverse). for Bax, ATG CGT CCA CCA AGA AGC TGA (forward) and AGC AAT CAT CCT CTG CAG CTC C (reverse); for Bcl-2, TTC GCA GCG ATG TCC AGT CAG CT (forward) and TGA AGA GTT CTT CCA CCA CCG T (reverse). Primers for GAPDH as the reference gene were GGT CGG AGT CAA CGG ATT TG (forward) and ATG AGC CCC AGC CTT CTC CAT (reverse).

### 3.9. Western Blot Analysis

M21 cells were treated with Matrine at a concentration of 0.2, 0.4 and 0.8 mg/mL for 48 h. Protein was abstracted, and Western blotting was performed, as previously described [53]. Protein concentrations of cell lysates were determined using the Bradford method. Briefly, certain quantized proteins were separated in SDS-PAGE and, then, were transferred to polyvinylidene difluoride (PVDF) membrane. After blocking at room temperature, membranes were incubated with different primary antibodies before being visualized and photographed. The antibodies used in the experiments were: anti-PTEN, anti-PI3K, anti-Akt1, anti-Akt2, anti-phosphor-Akt^ser473^, anti-p21^Cip/WAF1^, anti-cyclin D1, anti-ERK1, anti-ERK2, anti-phosphor-ERK1/2^pY204^, anti-Bcl-2, anti-Bax and anti-GAPDH, which were obtained from Epitomics (Burlingame, CA, USA). Anti-Akt 3 and anti-phosphor-Akt^Thr308^ were purchased from Santa Cruz (Dallas, TX, USA).

### 3.10. Cell Transfection

Cells were seeded in 96-well or 6-well plates before transient transfection, as described previously [53]. At indicated time points, cells were transfected with PTEN-specific siRNA by using Lipofectamine™ 2000 Transfection Reagent (Introvigen, Carlsbad, CA, USA), according to the manufacturer’s instructions. Then, the cells were treated with Matrine at indicated concentrations, and the controls were treated with medium in equal volume for 48 h. SiRNAs were synthesized by Introvigen and targeted the following cDNA sequences: for PTEN, 5′-GTA TAG AGC GTG CAG ATA A-3′. M21 cells with transient transfection siRNA were harvested for MTT assay and apoptosis assay.

### 3.11. Statistical Analysis

All experiments were performed in triplicate and were repeated at least three times. The results are given as mean values ± SD. Statistical significance was performed by using the one-way analysis of variance (ANOVA) test. The significance level was set as * *p* < 0.05, ** *p* < 0.01 and *** *p* < 0.001.

## 4. Conclusions

In conclusion, we evaluated the antitumor potential and investigated the molecular mechanisms of a natural product, Matrine, in melanoma ^V600E^BRAF harboring M21 cells. Matrine induced cell proliferation inhibition, cell cycle arrest and apoptosis in M21 cells. Matrine regulated both the caspase and mitochondrial pathways to trigger apoptosis. Furthermore, Matrine is a promising antitumor candidate with its remarkable anti-tumor efficacy and intended PTEN activation mechanisms.

## Figures and Tables

**Table 1:** IC_50_s * of Matrine in various cell lines.

Cell lines	Tissue origin	Matrine

		IC_50_ ± SD (mg/mL)
**M21**	Melanoma	0.769 ± 0.28
**MDB-MA-231**	Breast carcinoma	2.758 ± 0.19
**MCF-7**	Breast carcinoma	1.405 ± 0.35
**HCT116**	Colon carcinoma	1.242 ± 0.17
**RPE**	Human retinal pigment epithelium	>5

* IC_50_ is the concentration (mg/mL) that reduced cell numbers to 50% relative to vehicle-treated cells. IC_50_s were determined from experiments at least thrice, independently, and presented as the mean ± SD.
